# Roles of differently polarized macrophages in the initiation and progressionof pancreatic cancer

**DOI:** 10.3389/fimmu.2023.1237711

**Published:** 2023-08-11

**Authors:** Peter Storz

**Affiliations:** Department of Cancer Biology, Mayo Clinic, Jacksonville, FL, United States

**Keywords:** pancreatic cancer, PDA, macrophages, inflammatory, M1, M2, AAM

## Abstract

During development of pancreatic cancer macrophage-mediated inflammatory processes and the formation of cancerous lesions are tightly connected. Based on insight from mouse models we provide an overview on the functions of classically-activated pro-inflammatory and alternatively-activated anti-inflammatory macrophages in the initiation and progression of pancreatic cancer. We highlight their roles in earliest events of tumor initiation such as acinar-to-ductal metaplasia (ADM), organization of the fibrotic lesion microenvironment, and growth of low-grade (LG) lesions. We then discuss their roles as tumor-associated macrophages (TAM) in progression to high-grade (HG) lesions with a cancerous invasive phenotype and an immunosuppressive microenvironment. Another focus is on how targeting these macrophage populations can affect immunosuppression, fibrosis and responses to chemotherapy, and eventually how this knowledge could be used for novel therapy approaches for patients with pancreatic ductal adenocarcinoma (PDA).

## 1 Introduction

Pancreatic ductal adenocarcinoma (PDA) has one of the highest fatalities. Due to a lack of early detection markers, PDA is usually diagnosed at a late stage when it already shows aggressive local invasion and metastasis, as well as high resistance to chemo- and radiotherapy (1, 2). Characteristic for PDA is the desmoplastic stroma, an immunosuppressive microenvironment that forms a dense barrier which protects tumor cells from immunologic elimination and prevents penetration of chemotherapeutics (3). The stroma surrounding pancreatic lesions mostly consists of proliferating fibroblasts that deposit collagen, fibronectin and other extracellular matrix components, but also produce cytokines and chemokines to crosstalk with lesion cells and immune cells (4, 5). Immune cells in the lesion stroma are mainly regulatory T cells, myeloid derived suppressor cells and macrophages, while other cells, such as CD8+ T cells, which could act anti-tumorigenic, are largely excluded (6, 7). The PDA microenvironment not only blocks protective immunosurveillance (7), but also can act as a niche for cancer stem cells that can drive therapy resistance and recurrence (8). Since tumor immune infiltrates in pancreatic cancer increase inflammation and disease progression rather than contribute to disease eradication, immune gene or inflammatory signatures in the microenvironment allow predicting prognosis in patients (9, 10).

Animal studies suggest that different macrophage populations have key roles in all stages of PDA development by cross-talking with a variety of cell types. Macrophages show high plasticity and can display a range of activation states. The two extreme polarization phenotypes that have been described *in vitro* are classically-activated, M1-polarized, pro-inflammatory macrophages (M1, IM) and alternatively-activated, M2-polarized, anti-inflammatory macrophages (M2, AAM). For simplification, we will use these terms (M1 and M2) although there is some debate in the filed as to how closely these states match what has been identified *in vivo*.

Initiation of pancreatic lesions and the formation of the surrounding microenvironment requires activation of different macrophage populations. Pro-inflammatory macrophages (M1) have a major function in initiation of pancreatic lesions (11, 12), while alternatively-activated macrophages (M2) are involved in fibrinogenesis, resolution of inflammation, generation of an immunosuppressive environment and lesion growth (13, 14). However, in established tumors M1 promote Th1 (T helper type 1; CD4+ effector T cells) responses such as proliferation of cytotoxic (CD8+) T cells, and are involved in tumor rejection via release of pro-inflammatory cytokines and reactive oxygen species, while M2 promote tissue remodeling, immune regulation, tumor progression and metastasis (15–17). Therefore, although tumor-associated macrophages (TAM) represent a heterogenous spectrum of polarization types (18, 19), the majority share phenotypical and function features with M2 macrophages (16).

Advances in modeling PDA using genetically engineered mice have dramatically changed our view of pancreatic carcinogenesis (20, 21). The following paragraphs will summarize knowledge from animal models indicating how different macrophage polarization types contribute to PDA formation. We will also discuss approaches to target or reprogram macrophage populations with the net effect to generate a more pro-inflammatory, anti-tumorigenic environment that decreases the desmoplastic reaction and allows more efficient targeting of PDA cells.

## 2 The lesion progression model for pancreatic ductal adenocarcinoma

Inflammation and increased immune cell infiltration is a common feature of most behavioral risk factors for human pancreatic cancer, including alcohol-caused pancreatitis, obesity and smoking (2, 22, 23). Numerous animal studies demonstrated that in response to pancreatitis or after local inflammation, acinar cells undergo a reprogramming to a progenitor-like cell type with duct-like features that is highly-sensitized to malignant transformation (2, 11, 12, 24–26). This process is termed acinar-to-ductal metaplasia (ADM), resulting in earliest pancreatic lesions (24). ADM lesions, if they originate from normal cells can reverse to acinar cells once pancreatic inflammation is resolved and can contribute to pancreas regeneration (27–29). However, ADM cells with oncogenic mutations in KRAS or aberrant epidermal growth factor receptor (EGFR) signaling progress to low-grade pancreatic intraepithelial neoplasia (LG-PanIN) which are precursor lesions for PDA, or other types of pancreatic lesions (2, 25, 27).

In animal models in which oncogenic KRAS is expressed in pancreatic or acinar cells, ADM and PanIN formation usually occurs focally, suggesting lesion initiation via additional, non-genetic factors. Both the ADM process, as well as the progression of these lesions is tightly linked to a crosstalk with different macrophage populations. Inflammatory and alternatively-activated macrophages have been shown to secret factors that induce ADM (12, 30), but also factors that mediate lesion growth (12, 14, 30, 31). Progression from ADM to LG-PanIN then initiates a reverse crosstalk, in which lesion cells produce factors that initiate a polarization switch to an alternatively-activated macrophage type (14). M2 once present at lesions can activate pancreatic stellate cells (PSCs) to generate the fibrotic barrier and desmoplastic stroma around lesions (32). Other functions of these AAM are to provide factors that stimulate lesion growth and EMT-like structure collapse (32). Below, we will discuss in more detail these molecular events driving occurrence and progression of LG lesions, which were mostly obtained from mouse *in vivo* or explant organoid culture data.

Eventually, LG-lesions progress to cancerous pancreatic high-grade (HG) lesions such as PanIN3 (carcinoma *in situ*) and PDA (33), and both M1 and M2 macrophages contribute to the tumor-associated macrophage population, whereby M2-polarized AAM are a majority in the tumor microenvironment (13, 17). At these later stages of tumor development M2 macrophages are crucial for organizing the fibrotic tumor microenvironment and immune evasion. This also will be discussed in more detail below, using available insight from mouse models.

## 3 Classically activated inflammatory macrophages and their role in initiation of precancerous lesions

**Figure 1:**
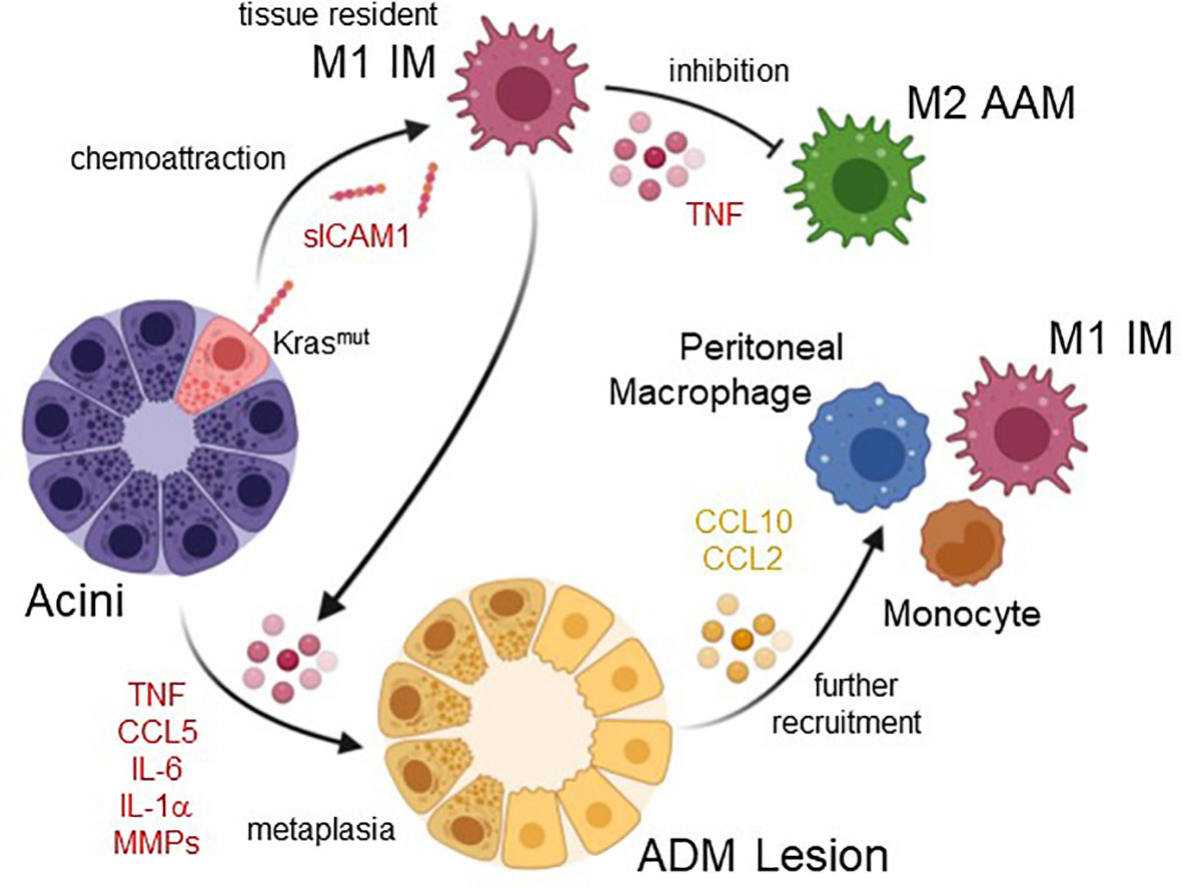
Roles of inflammatory macrophages in acinar cell metaplasia and formation of ADM lesions. Acinar cells with oncogenic Kras mutations upregulate expression of ICAM1, which as a shedded, soluble form (sICAM1) can attract inflammatory M1 macrophages (IM). These most likely originate from tissue resident populations. IM M1 once present at acini produce factors that inhibit alternatively-activated M2 macrophages (AAM), but also factors that drive acinar cell metaplasia to a duct-like phenotype. This acinar-to ductal metaplasia or ADM is driven by the IM secreted cytokines/chemokines TNF, CCL5, IL-6, and IL-1α, or by MMPs. Resulting ADM lesions are the earliest lesions that have been implicated in inducing the formation of pancreatic cancer. ADM can secrete molecules such as CCL2 and CCL10, which have been implicated in further recruitment of macrophages and monocytes. Created with BioRender.com.

M1-polarized inflammatory macrophages are characterized by high expression of pro-inflammatory cytokines, and increased production of reactive oxygen species (ROS) and nitrogen species (15, 16), and accumulating data suggest that they are main drivers of ADM *in vivo* (**Figure 1**). This classically-activated macrophage population in the mouse pancreas has been characterized by expressing F4/80, iNOS, IL-13Rα1, pY701-STAT1 and IRF5 as markers and by secreting various cytokines and chemokines including tumor necrosis factor α (TNFα), CCL5/RANTES, CCL2/JE, CCL3, CCL4, CXCL10/IP-10, IL-6, and IL-1ra (12–14).

ADM can be initiated by the M1-secreted factors TNFα, CCL5/RANTES, IL-1α and IL-6 (12, 34). A common underlying signaling event that these factors are activating in acinar cells is the increase in mitochondrially-generated reactive oxygen species (mROS) (35), which then drive ADM through transcriptional upregulation of EGFR and its ligands (36). It should be noted that oncogenic KRAS by itself is a weak inducer of ADM in *ex vivo* (explant) studies, also by increasing intracellular levels of mitochondrial oxidative stress and expression of EGFR (36). However, its effects are potentiated when M1-secreted factors are present (10), suggesting macrophage-induced signaling as a signal amplifier of KRAS autocrine signaling (34). Consequently, it was shown in *in vivo* studies that M1 macrophage infiltration in context of chronic pancreatitis or inflammation when induced by high fat diet can act synergistically with *Kras* mutations to induce ADM, and to accelerate development of PDA (26, 37).

Other M1 IM-induced events contributing to formation and progression of PDA are the initiation of changes in the ADM lesion microenvironment, which include effects on other immune cells and components that regulate extracellular matrix organization. For example, inflammatory macrophages produce matrix metalloproteinase 9 (MMP9) (38–41). During tumor initiation, signaling induced by M1-polarized macrophages also upregulates expression of MMP3, MMP7, MMP9, MMP10 and MMP13 in acinar cells (12), and MMP9 has been demonstrated to drive the ADM process via microenvironment remodeling (11, 12, 31). MMP9 is essential for angiogenesis and expansion of tumor lesions in pancreatic cancer (42), and both are mediated through MMP-regulated degradation of basement membrane and extracellular matrix, as well as release of matrix-bound growth factors (43). At earliest stages of lesion initiation acinar cells and pro-inflammatory macrophages also secrete factors that stabilize the M1 polarization phenotype or block M2 gene expression. These include acinar cell produced GM-CSF (12), which is associated with the M1 polarization process (44, 45), and M1-produced TNFα, which *in vitro* can block M2 gene expression (46). Another role for M1-polarized IMs may be in driving DCLK1+;CD133+ pancreatic cancer stem cell formation, which occurs in ADM and LG lesions at an early stage of tumor development (47–49). IL-6, an M1 macrophage secreted factor, has been shown to select for the CD133+ “stem-like” population by reprogramming metabolic pathways in pancreatic lesion cells, and to alter the pancreatic tumor microenvironment (50).

The origins of the pro-inflammatory macrophages that drive tumor initiation in the pancreas are not fully understood. While it is well documented that in KRAS-driven mouse models the presence of inflammatory macrophages increases rapidly in lesion areas, it is still unclear if they originate from a tissue resident population (51), from tissue infiltrating bone marrow-derived blood monocytes, from peritoneal populations (52), or both. The normal mouse adult pancreas has a low abundant self-maintained heterogenous tissue resident population of macrophages (53), including M1 type macrophages at the islets and mostly AAM in the exocrine pancreas (54, 55). During tumor development, expression of oncogenic KRAS in acinar cells generates an inflammatory microenvironment (56). This can be achieved through KRAS-induced expression of ICAM-1, which in its soluble form can act as a chemoattractant for classically-activated, inflammatory (but not alternatively-activated) macrophages, leading to focal inflammation (11). Thus, it seems likely that this initiating population is recruited locally from tissue resident macrophages (51). However, the rapid increase in population density suggests that further macrophages are also recruited to the pancreas from external sources such as peritoneal macrophages or circulating blood monocytes (57). This is supported by data showing that CXCL10, which is produced by ADM and LG lesions during the early phase of murine pancreatic cancer development, mediate the chemoattraction of additional M1macrophages to the pancreas, enhances their proliferation and maintains their inflammatory identity (58). Moreover, for obesity, one of the risk factors for PDA (59, 60), it was shown that it augments and sustains M1 IM-driven ADM through chemoattraction of bone marrow-derived monocytes via CCL2 (61).

In summary, pro-inflammatory, classically-activated macrophages are the major immune cell population driving the formation of ADM lesions, and, in presence of KRAS mutations, prime lesion cells for further progression.

## 4 Roles of alternatively-activated macrophages in lesion progression

**Figure 2:**
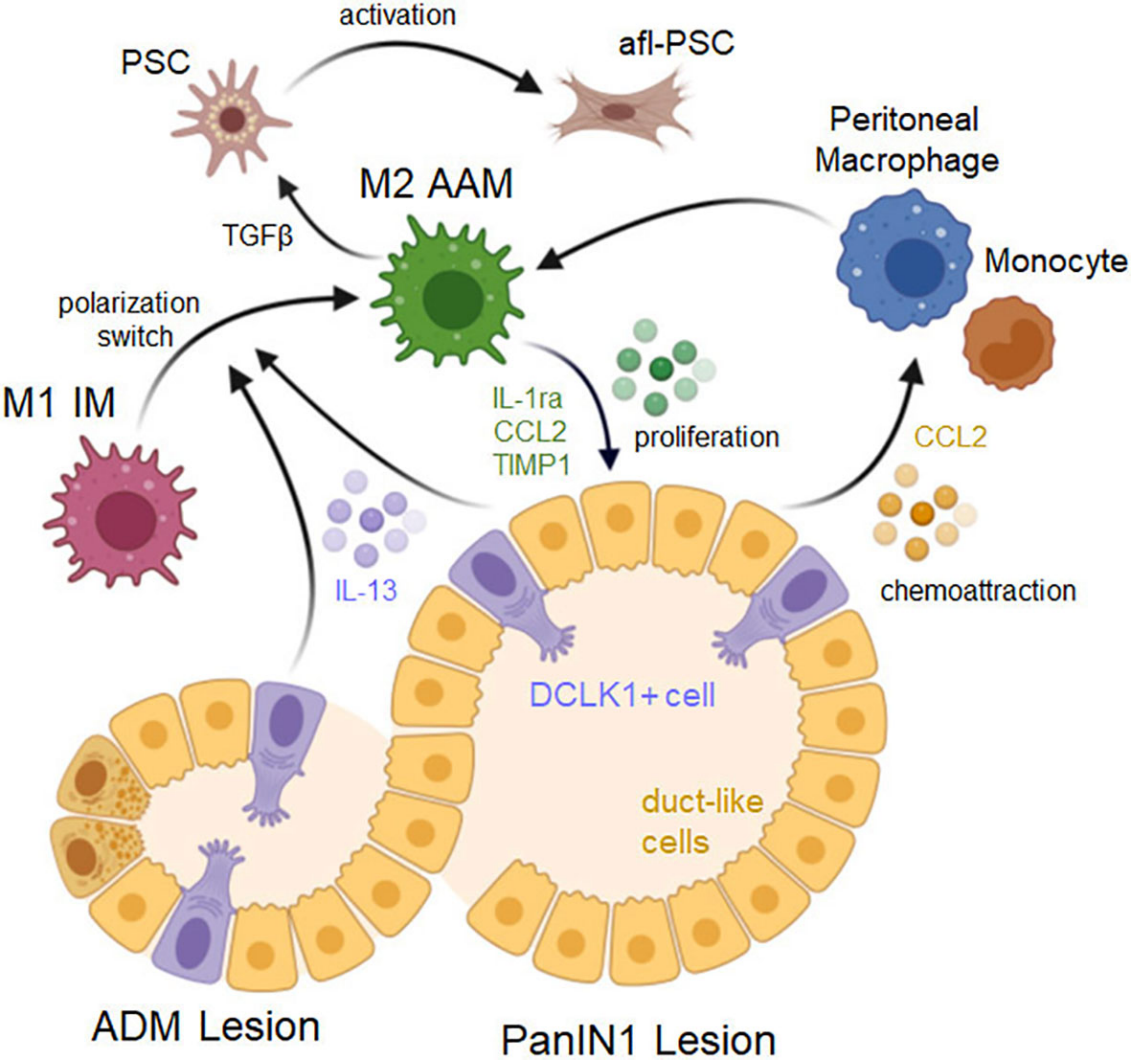
Signaling driving a macrophage phenotype switch and lesion progression. ADM lesions consist of duct-like cells that originated from acinar cells as well as a small fraction of DCLK1 positive (DCLK1+) cells that originate from duct-like cells. Both, DCLK1+ cells and duct-like cells (but to a lesser degree) secrete IL-13, which induces a polarization switch in macrophages from an inflammatory to an alternatively-activated M2 phenotype. Resulting cells express M2a markers and induce lesion cell proliferation via secretion of IL-1ra, CCL2 and TIMP1, and induce fibrosis by activating pancreatic stellate cells (PSCs) via TGFβ. PanIN lesion cells also produce CCL2 which can act auto-stimulatory to enhance lesion growth, but also serves as a chemoattractant for peritoneal macrophages or monocytes. Created with BioRender.com.

While inflammatory M1 macrophages are the dominant macrophages population at ADM lesions, further progression of lesions goes along with increased presence of alternatively-activated macrophages (AAM, M2) (**Figure 2**) (14), which have low cytotoxic functions and high tissue remodeling activity (62). As for the M1 IMs that drive ADM, the origin of the M2 AAM population at early stages of tumor formation is still in discussion. While it was suggested that M2 macrophages may infiltrate the pancreas (63), recent work indicates that these AAM at the earliest stages of tumor development may originate from inflammatory macrophages, supporting a polarization shift over chemoattraction (14).

In ADM and LG-PanIN, DCLK1+ cells and lesion cells are important regulators of macrophage polarization. Both cell types secret IL-13 to induce a shift from M1 to M2 polarization (14). At this stage of cancer development IL-4 is not detected, which is a major difference to pancreatitis, in which a M1/M2 switch is induced mostly by IL-4 secreted from pancreatic stellate cells (29, 64). However, it was shown that in established PDA, cancer cells can also be a source of IL-4 to further increase the alternative macrophage population (65, 66). At this stage it is also likely that the phenotype of M2 macrophages is additionally controlled by CD4+ T helper type 2 (Th2) cells that express IL-4 and IL-13, and counteract the Th1 cell responses, as observed in other cancers (16, 67).

The importance of a switch from M1 IM to M2 AAM for the development of PDA has been demonstrated by blockage of CXCL10/CXCR3 signaling on M1, which shifts macrophage populations to the tumor promoting M2 AAM phenotype and enhances progression of pancreatic precancerous lesions (58). While macrophage polarization is regulated by a multitude of transcription factors and regulators, the interferon-regulated factors (IRFs) IRF4 and IRF5 seem to be critical. For example, IRF4 negatively-regulates toll-like receptor (TLR) signaling and drives M2 polarization (68), while a decrease in IRF4 and switch to expression of IRF5 drives M1 polarization (13, 58, 69).

The M2 AAM (YM1+ AAM) macrophage population at PanIN lesions has been characterized by expressing chitinase-like protein 3 (CHIL3) or YM1 as most characteristic marker, as well as other markers such as Arginase 1, FIZZ1, IL-1ra, IL-10, CD206/MRC1, TREM2 and C1QB (13, 32, 70, 71). According to the classical/alternative model of macrophage activation, based on above markers this YM1+ AAM population in mice is most close to the M2a subtype of M2 (13, 32, 72, 73). YM1+ AAM stimulate multiple immunosuppressive and cancerogenic effects, and striking results can be obtained in mice, when this population is targeted. This can be achieved with either neutralization of IL-13 (14), or with pomalidomide, a thalidomide analog that has been developed for hematologic cancers and induces a phenotype switch from an alternatively-activated M2 to an inflammatory M1 phenotype (13). Reducing or blocking YM1+ AAM has several effects on early lesion cells and cells in the lesion environment. YM1+ AAM secrete factors such as CCL2, which can drive ADM (30), as well as TIMP1, IL1-ra and CCL2 to promote the proliferation of precancerous lesions cells, and their depletion leads to significant decrease in lesion growth (14, 32).

Another characteristic of mouse pancreatic cancer is the formation of DCLK1+ cells at the stage of precancerous lesions, of which a subpopulation is believed to function as PDA stem cells (47–49), while pancreatic Tuft cells (which are also positive for DCLK1) seem to act inhibitory with respect to tumor formation (74, 75). Epithelial-to-mesenchymal transition (EMT) can induce a stem cell phenotype that facilitates recurrence of therapy resistant tumors and distant metastasis (76). TGFβ1, for which YM1+ AAM are main producers in developing PDA, can induce EMT signaling in lesion cells (32), suggesting that YM1+ AAM may have a role in initiating early dissemination of cells from lesions. However, it needs to be rigorously tested if this is related to an increase in circulating pancreatic DCLK1+ cells.

Most striking after targeting the YM1+ AAM population are effects observed on fibrinogenesis and on the attenuated immune response in lesion areas (13, 32). Fibrosis at early lesions is driven by pancreatic stellate cells (PSC) (77) and many functions that have been attributed to activated PSC may be due to presence of YM1+ AAM. YM1+ AAM are major drivers of fibrinogenesis by activating quiescent PSC (32). This is mediated by YM1+ AAM-produced TGFβ1, which drives expression of fibrosis markers such as smooth muscle actin (SMA) (32). Activated stellate cells then can auto-stimulate via CXCL12 to drive further proliferation (32), but in a feedback loop also stimulate cytokine production in macrophages (78). Like activated, fibroblast-like PSC (afl-PSC) at early lesions, myofibroblast-like fibroblasts are increasingly abundant during progression from PanIN to cancer (78). Fibroblast populations at full-blown PDA are more heterogenous and range from myofibroblast-like CAFs (myCAFs) to other, less abundant fibroblast populations with distinct functions, such as inflammatory CAFs (iCAF) and antigen-presenting CAFs (apCAF) (79, 80). At this point it is not fully clear if the activated fibroblast-like cells that derive from pancreatic stellate cells have the plasticity to develop into myCAFs (81). However, depletion of the YM1+ AAM population using Pomalidomide also dramatically decreased the desmoplastic reaction in established syngeneic tumors (13, 32), and therefore may also be a valuable target to decrease fibrosis driven by myCAFs in established tumors.

Infiltrating immune cells, such as tumor-infiltrating lymphocytes, mast cells and macrophages impact pancreatic tumor development and progression by contributing to an immunosuppressive microenvironment (82). For example, M2 AAM restrain the inflammatory response by producing the cytokines IL-10 (5) and TGFβ1 (32), but also inhibit T cell responses (83). Consequently, depleting YM1+ AAM in KC (LSL-Kras^G12D/+^;p48^cre^) mice, an animal model for precancerous lesions, or in mice with syngeneic KPC (LSL-Kras^G12D/+^;Trp53^R172H/+^;PdxCretg/+) tumors increases presence of activated CD4+ and CD8+ effector T cells and shifts the lesion microenvironment from immuno-suppressive to immune-responsive (13). Similar effects to direct targeting of YM1+ AAM were observed when mice are vaccinated with TGFβ-derived peptides. After vaccination, tumors showed reduced fibrosis as measured by a decrease in myCAFS and led to increased presence of CD8+ T cells (84). Thus, targeting this AAM population or its downstream effectors holds great promise for pancreatic cancer therapy. However, it should be noted that while the importance of YM1+ AAM in mouse tumorigenesis becomes increasingly clear, the human counterpart to this population is not fully defined, yet.

## 5 Tumor-associated macrophages in pancreatic cancer

Increased presence of tumor-associated macrophages (TAMs) in full-blown PDA is associated with increased metastasis and poor outcome (85). The majority of TAM in pancreatic cancer patients were described to share phenotypical and functional features with M2 type macrophages (16). However, it was demonstrated for freshly isolated human TAM that they are not only composed of macrophages that were positive for CD163 and IL-10, representing the alternatively-activated M2 phenotype, but also contained macrophages positive for TNFα and IL1β and HLA-DR, thus representing an inflammatory M1 macrophage population (86). This heterogeneity of macrophages and the relative composition of polarization types may contribute to the formation of different human (basal or classical) subtypes of PDA (87).

**Figure 3:**
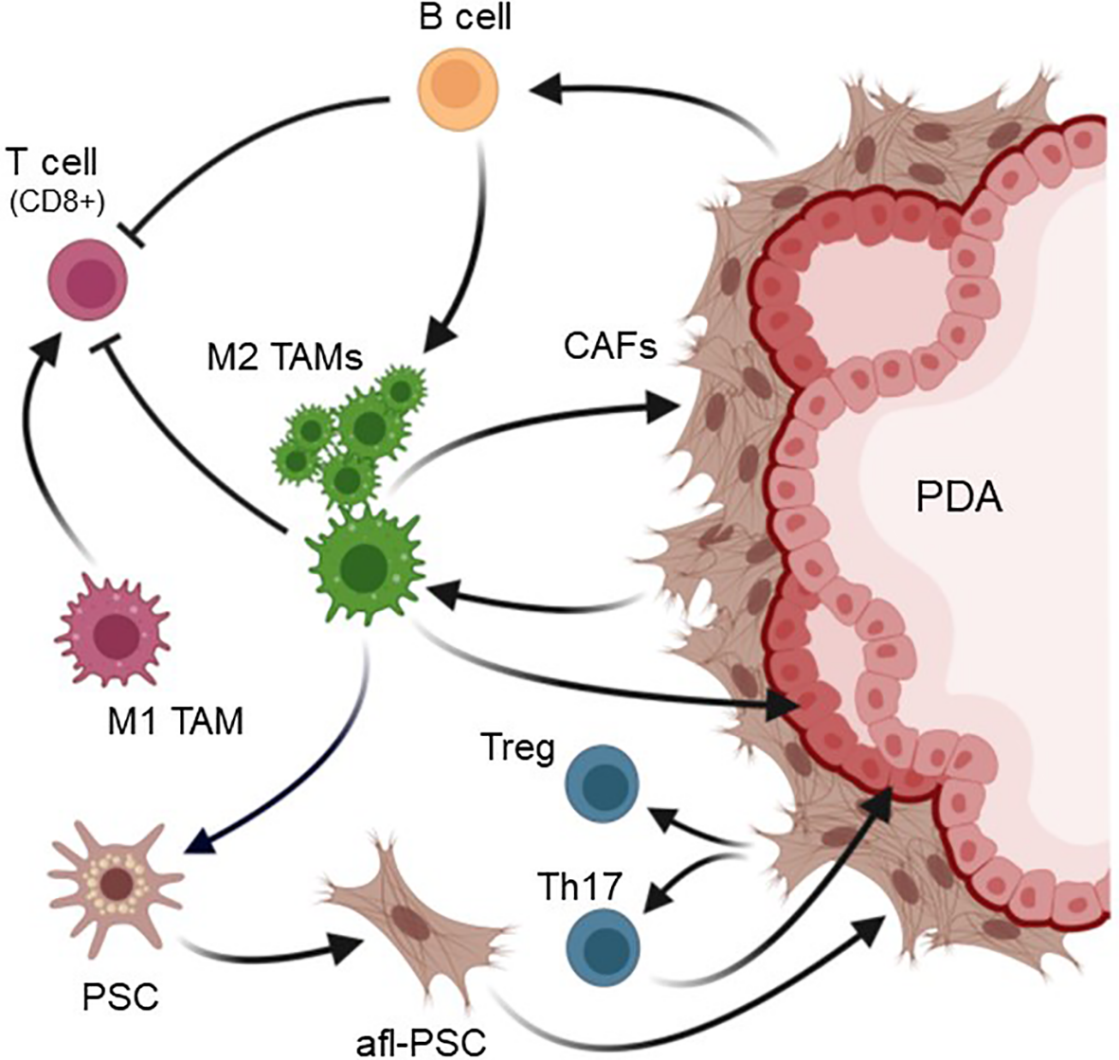
Crosstalk of TAM and other cell types in the PDA microenvironment. In PDA the majority of tumor-associated macrophages (TAMs) are M2 polarized. M2 TAMs generate an immunosuppressive and tumor-promoting microenvironment. They activate PSC to become activated fibroblast-like cells (afl-PSC). These cells together with other cancer-associated fibroblasts (CAFs) contribute to the fibrotic layer around pancreatic cancer cells. In turn, CAFs can stimulate M2 TAMs and contribute to the presence of B cells and CD4+ Th17 and Treg cells. Both B cells and M2 TAMs inhibit cytotoxic (CD8+) T cells, resulting in an immunosuppressive environment. Additionally, M2 TAM release factors that promote the expansion of cancerous lesions. Created with BioRender.com.

In mice, TAM in pancreatic tumors are also a heterogenous population consisting of M1 (~15%) and M2 (~85%) subtypes (88), with a large population that expresses M2a markers similar of the YM1+ AAM macrophages in the precancerous phases (89) and, like this population at early lesions, these YM1+ TAM have been implicated in exhibiting a pro-fibrotic transcriptional and immunosuppressive profile (13). While M1 are more corelated to early-stage cancer and at a later stage are seen as acting more anti-tumorigenic, an increase in M2 is more corelated with a progressed phenotype, in which inflammation is suppressed (**Figure 3**).

In the KPC model TAM have heterogenous origins and can derive from populations that are already present at precancerous lesions (51), but also result from newly recruited infiltrating population (11, 90). For example, during cancer progression bone marrow-derived (BMD) myeloid cells, including immature myeloid cells and macrophages, have been shown to accumulate at the primary tumor site and contribute to the establishment of a tumor-promoting microenvironment (63, 91). CCL2, which is also expressed by LG lesion cells (12), may further contribute to BMD monocyte and macrophage infiltration into tumors (92), as previously observed in human cancer (93). Moreover, further recruitment of inflammatory macrophages into pancreatic tumors is mediated by several cytokines. These include CSF1, a chemotactic factor for macrophages, and a major growth factor for TAM, as well as vascular endothelial growth factor (VEGF) which has been described to recruit peritoneal macrophages that express VEGF receptors (94).

Tumor-associated macrophages can adopt different activation states, and macrophage polarization in pancreatic cancer is a dynamic event. It has been shown to be dependent on the balance between TNFα signaling, which reduces M2 gene expression, and factors that induce a M1 to M2 polarization (46). The high presence of the M2 polarization type in pancreatic cancer is mediated by multiple factors secreted from cancer and stromal cells. For example, lesion cells produce IL-13, and PSC and cancer cells can produce IL-4, both factors that drive M1 to M2 polarization (14, 64). IL-13 can also be secreted from eosinophils that are co-recruited with inflammatory monocytes (46). In addition, M2 subtypes can release IL-10, an anti-inflammatory cytokine, to further establish the M2 TAM population (95). Another factor that favors activation and presence of M2 macrophages is lactate efflux from PDA lesions cells (50).

TAMs in PDA are indicative for poor survival prognosis. This is mostly due to the M2 population of TAMs, which promote angiogenesis, reorganization of the extracellular matrix, and correlate with larger tumor size (49, 61, 92).They also are associated with emergence of cancer stem-like cells and with local recurrence and metastasis (17, 63, 96). Moreover, M2-polarized TAM act immunosuppressive and render immunotherapy ineffective, but also contribute to resistance to chemotherapy (63). For example, it was shown that TAM *in vitro* can have a drug scavenging effect and rapidly metabolize the anti-cancer drug gemcitabine, and in tumor bearing KPC mice the pharmacological depletion of TAM improves the therapeutic response to gemcitabine (97).

## 6 Modulation and reprogramming of macrophages as strategies for pancreatic cancer prevention and therapy

**Figure 4:**
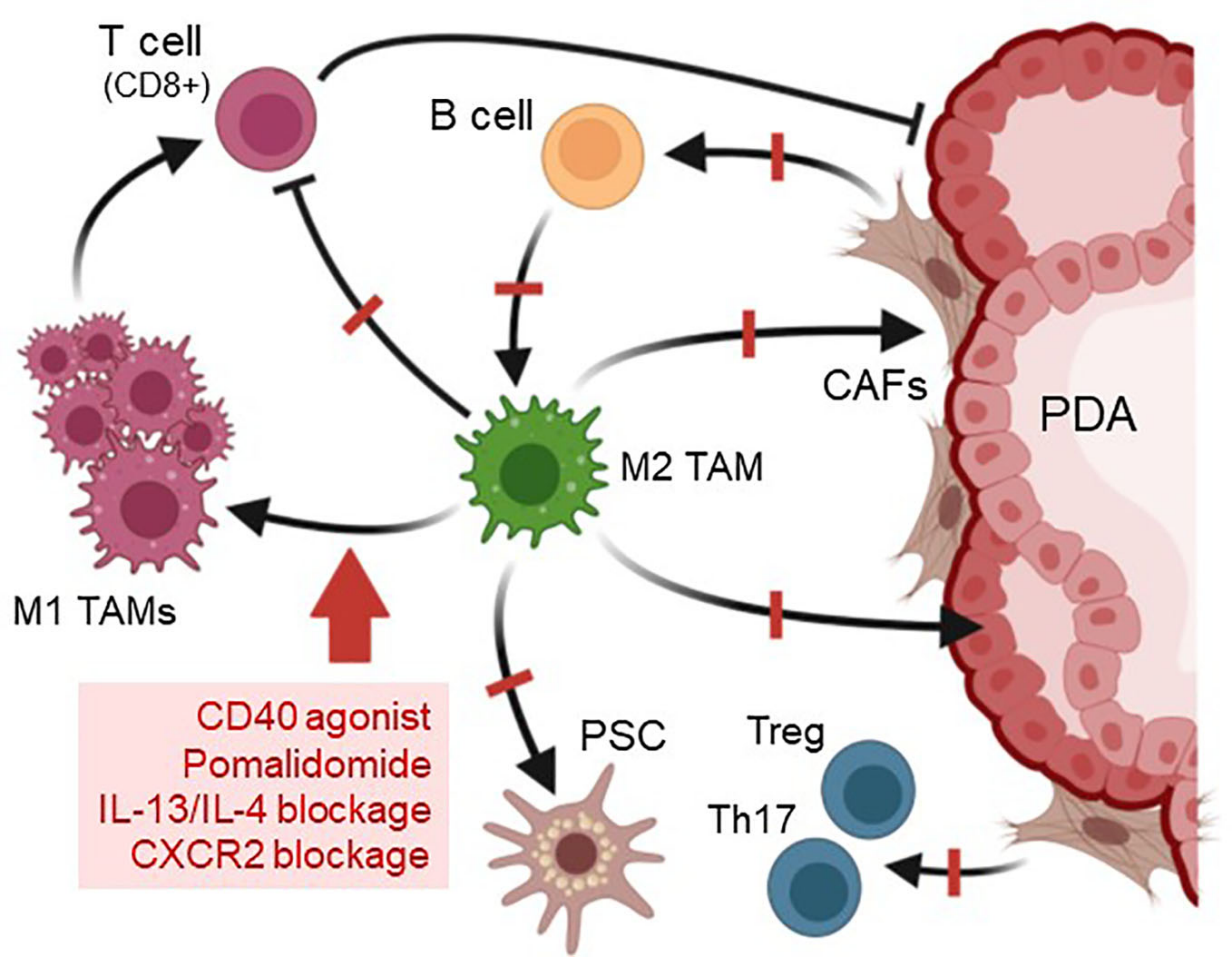
Strategies to target M2 TAM and predicted outcomes. Due to their pivotal role in organizing the pancreatic lesion microenvironment, one option to increase the efficiency of chemotherapy in PDA is targeting M2 TAMs. This can be achieved by treatment with CD40 agonists, blockade of CXCR2 or of IL-13/IL-4, or by drugs such as Pomalidomide, which affect the balance of IRF4/IRF5 transcription factors. Targeting of M2 TAMs with these methods leads to a polarization switch to pro-inflammatory M1 macrophages, which activate cytotoxic (CD8+) T cells. Other effects observed after depletion of M2 TAMs in mouse models for PDA are a significant decrease in fibrosis and slower lesion growth. Created with BioRender.com.

Established pancreatic cancers develop an immunosuppressive, fibrotic microenvironment, which forms an efficient barrier around tumor lesions that sequesters T cells and prevents efficient delivery of chemotherapeutic agents (98). Stromal depletion is a key strategy to decrease this fibrotic barrier surrounding pancreatic lesions and to relieve vessel compression to increase drug delivery to hypovascular tumors (3, 99). This can be achieved with relatively non-specific agents such as nab-paclitaxel, which targets both stromal and tumor cells, and in combination with Gemcitabine mediates tumor regression (100). However, it also has been shown that the direct targeting of stromal fibroblasts in mouse PDA can result in accelerated tumor progression (101, 102), most likely because the rapid debulking of fibroblasts allowed expansion of tumor lesions or because different subtypes of cancer associated fibroblasts (CAFs) can have tumor restraining or tumor-promoting roles (103). Therefore, targeting the M2a-like YM1+ AAM macrophage population that organizes fibrosis by activating stromal fibroblasts and promoting immune cell exclusion, could be a more efficient option (**Figure 4**). Another benefit of such an approach may be that it induces responsiveness of PDA to immune checkpoint inhibition (104, 105). Immune evasion and inhibition of T cell anti-tumor response in mouse PDA is mediated by upregulation of programmed cell death-ligand 1 (PD-L1) in tumor cells (106), but immunotherapy approaches that target PD-1 so far were not successful for this cancer.

Altering the presence of macrophages can be achieved by targeting the differentiation of hematopoietic stem cells into macrophages by blocking colony stimulating factor 1 (CSF1) or its receptor, an approach which also synergized with checkpoint immunotherapy (107). Another key anti-cancer immunotherapy approach is to change the polarization type of M2 TAMs to M1 kill-type macrophages (108). The elegance of most approaches that induce such a shift in macrophage polarization is that they also increase the presence of activated T cells, which exhibit anti-tumor activity. For this, a promising strategy is the use of CD40 agonistic antibodies which shift TAMs to tumoricidal macrophages that rapidly infiltrate tumors and facilitated the depletion of tumor stroma (109). Moreover, presence of these inflammatory macrophages increased efficacy of gemcitabine in patients (109, 110). Increases in M1 macrophages and decreases in M2 can also be obtained after blockage of CXCR2 signaling (111). In addition, the inhibition of lactate efflux from PDA lesions cells to decrease activation of M2 macrophages, has been shown to increase CD8+ T cells and makes tumors more responsive to anti-PD1 therapy (50). Similar shifting to M1 as well as decreased fibrosis and increased T cell influx is observed in mouse models for precancerous lesions (KC model) and for cancerous lesions (KPC syngeneic tumors) with reagents such as the thalidomide analogue pomalidomide, which modulates the IRF4/IRF5 expression ratio to induce a M2 to M1 polarization switch (13, 58, 112). Moreover, in KC mice, targeting the YM1+ AAM population via neutralization of IL-13 showed promising results and decreased fibrosis and immunosuppression (13, 14). However, for both, POM and IL-13 neutralization, it needs to be further established if additional combination with chemotherapy is efficient to debulk tumors and to induce tumor regression.

## 7 Conclusions

A complex immunosuppressive tumor microenvironment is a hallmark of pancreatic cancer and strategies that aim to target stromal functions and to restore the immune responsiveness showed great promise to increase efficacy of conventional therapies (8). Understanding the origins and functions of macrophages that promote pancreatic tumorigenesis may be important for further optimizing therapeutic intervention, such as the blockage of macrophage influx into the pancreas or to induce a shifting in their polarization to a pro-inflammatory anti-tumorigenic phenotype.

YM1+ M2 macrophages due to their key roles in PDA development and progression could be the Achilles heel for PDA. We here highlighted the importance of YM1+ M2 macrophages for tumor development, progression and immune evasion, and discussed successful approaches to modulate or reprogram these cells. Key tasks now are to define the exact counterpart of this population in human tumors and to test if it can be targeted with similar means and outcome.

Another benefit to further characterize this macrophage population is that they may produce specific secreted markers or induce stromal cells to release molecules that suggest tumor development. This could lead to the discovery of circulating immunological markers that can serve as early detection markers, or as prognostic and predictive biomarkers which are needed to stratify patients for available treatments (113).

## Author contributions

PS wrote the manuscript.
